# Audit of Tracheostomy Care Practices in a Nigerian Tertiary Neurosurgical Intensive Care Unit According to Published Guidelines

**DOI:** 10.7759/cureus.50160

**Published:** 2023-12-08

**Authors:** Kenechukwu K Igbokwe, Reginald N Ononye, Daniel E Onobun, Ugochukwu C Ugwuanyi

**Affiliations:** 1 Neurological Surgery, Wellington Clinics Abuja, Abuja, NGA

**Keywords:** ventilator-acquired pneumonia, tracheostomy complicatons, care, neuro-critical care, quality improvement, tracheostomy care practices

## Abstract

Introduction: Chest infections are a frequently encountered problem in patients admitted to the intensive care unit (ICU), more so in tracheostomised patients. This study aimed to audit the tracheostomy care practices in patients with neurosurgical pathologies in the ICU of Wellington Clinics Abuja, a tertiary neurosurgical hospital in Nigeria.

Methods: We conducted a closed-loop audit with mixed methods involving analysis of 24 patients who had tracheostomy within the first two weeks of neurosurgical pathology at a tertiary neurosurgical hospital and semi-structured interviews to determine tracheostomy care practices among the primary caregivers - nurses, intensivists, and doctors.

Results: Of the 161 ICU admissions in the first cycle, 22 patients received tracheostomy, 16 met the eligibility criteria. At re-audit (second cycle), eight of 40 patients met the criteria. All the patients received open suctioning through a dual cannula-cuffed tracheostomy tube and had independent portable suction units. In the baseline audit (first cycle), suction catheters were reused for 12-24 hours in each patient and were stored in varying combinations of normal saline and Savlon antiseptic (5 mg of cetrimide (0.5% w/w) and 1 mg of chlorhexidine digluconate (0.1% w/w)). The frequency, technique, and assessment of the need for airway suctioning were inconsistent among caregivers interviewed. All 16 patients had at least one episode of pneumonia, 10 patients had a second episode, and two patients had > two episodes. One mortality was recorded directly attributable to the complications of pneumonia. While in the re-audit, with adherence to recommendations, three patients suffered one episode of pneumonia and only one had a second episode. No mortality was recorded.

Conclusion: A standard practice guideline was necessary for tracheostomy care in our ICU. In low-resource settings, stated recommendations such as single-use suction catheters and improved hygienic practices can reduce rates of pneumonia in tracheostomised patients.

## Introduction

Neurosurgical outcomes, either congenital or acquired, frequently require intensive care pre and/or post procedures. Clinical outcomes vary and are determined by a variety of factors related to the mechanism of insult [1,2]. Consequently, a combination of depressed consciousness levels, inability to protect the airway, disruption of natural defence barriers, and decreased mobility is associated with prolonged mechanical ventilation and stay in the intensive care unit (ICU) [2-4].

Pulmonary complications, such as chest infections, have been reported to be as high as 60% in these patients and associated with a poor prognosis [4-6]. Chest infections are common in ICU-admitted patients and tracheostomy is often used to provide respiratory support in patients with neurosurgical pathologies [4]. It is known to reduce the work of breathing, reduce anatomical dead space, and facilitate airway toileting, theoretically decreasing the susceptibility of ventilated patients to chest infection [7,8]. Tracheostomy, however, may have a long treatment period [7,8]. Therefore, care of tracheostomy in neurosurgical ventilated patients can be challenging and if performed by poorly trained personnel, or with poor standards employed, can lead to increased incidence of ventilator-associated pneumonia with its attendant morbidity and mortality [6,9].

We report the findings of an audit of the impact of tracheostomy care practices on the incidence of chest infection in neurosurgical patients of an ICU in a resource-poor setting, detail the recommendations in the appendices, and report the second cycle findings after the recommendations were employed.

## Materials and methods

This study was conducted in the ICU of Wellington Clinics Abuja, focusing on tracheostomy surgeries performed by a single consultant surgeon. All patients had dual cannula-cuffed tracheostomy tubes and independent suction units in open tracheal suction systems.

The study employed a mixed methods approach, involving a dual-cycle closed-loop audit, to audit tracheostomy care practices. The first cycle was the baseline audit and involved retrieving and analysing all patient records between January 2020 and June 2021 to identify patients with all forms of brain injury admitted to the ICU who received tracheostomy as a definitive airway. To ascertain tracheostomy care practices, semi-structured interviews and focused group discussions were conducted in English with primary caregivers, including nurses, doctors, and support staff. The eligibility criteria comprised all patients with brain injury admitted into the ICU who underwent tracheostomy as definitive airway management within the first two weeks of admission. The incidence and frequency of chest infections within this cohort were recorded. The tracheostomy care practices (Appendix 1) were also noted.

The second cycle (re-audit) used the same eligibility criteria as in the first cycle but with prospective data collection and was conducted between July and October 2021. It involved all consecutive eligible patients and implemented a multifaceted intervention strategy incorporating standards of tracheostomy care from published guidelines, as this has been shown to be the most appropriate approach to use when implementing change in performance in a clinical setting [10]. In this strategy, reviewed tracheostomy care practices were discussed in a multidisciplinary clinical meeting and matched against recommended guidelines by the faculty of the Intensive Care Society [11]. Recommendations were adopted and are detailed in the appendices (Appendix 2). Hospital management accepted and implemented the recommendations, while compliance to adherence was monitored. Regular education sessions for care providers were also instituted to illuminate indications for suctioning (Appendix 3).

The primary outcome measure was at least one episode of acute chest infection, evidenced by documented physical, radiological (where available), and laboratory findings, as well as other systemic inflammatory response syndrome (SIRS) indicators occurring 48 hours or more after tracheostomy, with or without chest radiographs, was the primary focus [12]. The secondary outcome measure was mortality directly attributed to chest infection and its complications. Simple descriptive statistics, including mean and standard deviation (SD), were used to analyse the data. All data entry and analysis were performed using Microsoft Excel 2016 (Microsoft Corporation, Redmond, WA).

## Results

In the first cycle, 16 of 161 cases were included for review based on the eligibility criteria. The demography consists of six adult females, seven adult males, and three children, with a mean age of 43.4 years (range of 11 to 80 years; median age of 34 years), while in the second cycle, eight cases of 40 patients were included. The demography consists of five adult males, one adult female, and two children, with a mean age of 36.75 years (range of eight to 75 years; median age of 31 years) (Table 1).

**Table 1:** Age and gender distribution of the patients in the study N: total number of patients; SD: standard deviation.

Characteristics	First cycle		Second cycle	
	N	%	N	%
Sex				
Female, n (%)	7	43.8	2	25
Male, n (%)	9	56.4	6	75
Age (years)				
<18 (%)	3	18.8	2	25
18-59 (%)	7	43.8	5	62.5
>60 (%)	6	37.5	1	12.5
Total, No. (%)	16	100	8	100
Mean (SD)	43.4 (21.3)		36.8 (19.3)	

Existing practice

There was a marked inconsistency in the frequency, technique, and assessment of the need for airway suctioning, including the requisite changes of the respiratory circuit when necessary. Suctioning was performed with catheters that were reused for 12-24 hours in each patient and were stored in varying combinations of normal saline and Savlon antiseptic (5 mg of cetrimide (0.5% w/w) and 1 mg of chlorhexidine digluconate (0.1% w/w)). A paucity of insurance coverage and thus prevalence of out-of-pocket healthcare financing resulted in severe resource constraints. This meant that catheters were used for longer periods in these patients. Furthermore, there were instances of suction catheter shortages at the pharmacy, further resulting in single suction catheters being reused an entire day and discarded only at the end of the day to manage supply. Care provider hand hygiene and use of sterile gloves were not in practice and patients were not pre-oxygenated prior to suctioning (Appendix 1). The following prophylactic strategies were, however, the standard: head elevation (30-40°), regular oral hygiene, regular aspiration of subglottic secretions, protocol for sedation, and enteral nutrition via nasogastric tubes.

Following the implementation of recommendations (Appendix 2) and monitoring of compliance, a focused group discussion in the second cycle with the nursing staff revealed improved consistency, marked adherence to the recommendations, and increased knowledge of tracheostomy care.

Incidence and frequency of chest infections

A review of all 16 cases in the first cycle revealed at least one episode of acute chest infection (100%), with each episode averaging nine days (six to 12 days). Ten (62.5%) patients suffered a repeat episode and two (12.5%) had multiple (>2) episodes of acute chest infection. One (6.25%) death was recorded directly attributable to the complications of pneumonia and sepsis in the background of brain injury. Other morbidities recorded included partial/complete obstruction of the inner lumen by thick dry mucous plug and bleeding within the respiratory tract.

**Figure 1:**
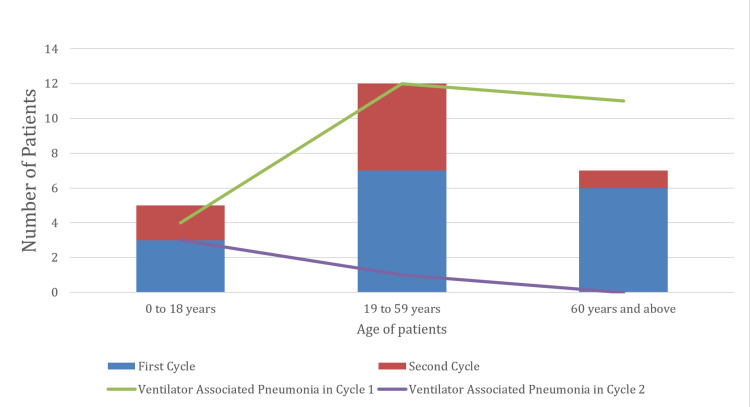
Chart comparing the incidence of chest infections within the age groups in both respective cycles

In the second cycle, we found a marked decline in the incidence and frequency of chest infections, with only three patients (37.5%) developing at least one episode of chest infection and one (12.5%) having two or more episodes. The average illness duration remained unchanged at nine days. There was no recorded (0%) mortality from chest infection and sepsis attributable directly to the complications of tracheostomy and its care (Table 2). We further compared the incidence of chest infection among the various age groups in both cycles (Figure 1).

**Table 2:** A summary of the primary and secondary outcomes (incidence of chest infection and mortality attributable directly to chest infection, respectively)

	At least 1 episode, N (%)	2 or more episodes, N (%)	Mortality, N (%)
First cycle	16 (100)	3 (18.8)	1 (6.3)
Second cycle	3 (37.5)	1 (12.5)	0 (0)

## Discussion

Tracheostomy is a life-saving procedure often indicated in patients requiring prolonged ICU admission [13]. In our ICU, it was found to be the second most common procedure performed averaging 12 per 100 patients. In patients with neurosurgical pathologies, the main indications for tracheostomy placement include failure to wean off from endotracheal tubes, predicted prolonged period of invasive mechanical ventilation, absence of protective airway reflexes, inability to maintain airway patency, and reduced/absent central respiratory drive [14,15]. The likelihood of receiving a tracheostomy increases significantly with age, the severity of neurological injury (Glasgow Coma Scale score </= 8, pupillary abnormalities), extracranial injury (particularly thoracic trauma), and early secondary insults (such as hypoxemia) [16]. In traumatic brain injury, non-neurological factors in the decision to perform a tracheostomy on a patient include age, concomitant thoracic trauma, and predicted difficult weaning from mechanical ventilation [16].

The care of tracheostomy was thought to be a major contributing factor to the high rates of chest infection observed in our ICU; this was proven by the audit, with a significant fall in incidence (100% to 37.5% and 18.75% to 12.5% with at least one episode and two or more episodes, respectively) recorded with standardization of tracheostomy care approach (Table 2). We also recorded a reduction in morbidity and mortality associated with tracheostomy in this patient group. Although our chest infection rates remained as high as those reported in the literature, our patient population is a particularly vulnerable group as indicated by the primary pathology [4,6]. We found the peak incidence of chest infection among the age group of 18-59 years, followed by the >60 years age group. Figure 1 summarises the incidence of chest infection by age group. Increasing susceptibility to chest infection with age and reduction in physiologic reserve could explain the higher rates in older compared to younger patients, as observed in the study [17,18]. These findings are consistent with the peak age of chest infections reported in the literature [4,5,17].

The commonest deviations from standards observed in our study were the reuse of suction catheters, poor hygienic practices around storage and handling, and poor handwashing techniques, and this was compounded by a lack of trained staff and resource limitations [12]. A study in Egypt reports a reduction in chest infections following the adoption of single-use suction catheters [19]. Airway suctioning was a frequently reported challenge in tracheostomy care; inconsistencies were found among the nursing staff regarding timing and frequency, contributing to increased morbidity associated with tracheostomy. This is not unique to our practice, and although the frequency of suctioning is ideally tailored to individual patient’s needs, some general principles exist [20]. We developed a checklist system to aid in standardizing the approach to airway suctioning in our ICU and implemented training and simulation activity for new/existing staff to re-emphasize and recognize the indications for airway suctioning (Appendix 3).

Education, training, and retraining were central to our intervention. Recommendations resulting in policy change around suction catheter use and written sustainable ICU policy (Appendix 2) for tracheostomy care within resource restriction are believed to have played a vital role in the marked improvement in our chest infection rates. These recommendations were developed and adapted to our practice from published guidelines [11]. Provider training for tracheostomy care has been noted to be important in maintaining standards and giving the provider the requisite knowledge relevant to confidence and comfort for the level of care that they are providing [21,22]. Hands-on and simulation training for tracheostomy have been proven to be effective and also give the provider confidence and comfort while handling the emergent care for the patient [22,23]. The impact of this has been seen in the results with the reduced number of episodes of chest infection and reduced mortality observed in the re-audit.

The presented results must be considered in the context of the limitations of this study. Being a retrospective study (first cycle), the lack of randomization in the design of the study inevitably hinders the generalisability of the conclusions that can be drawn. In addition, data regarding underlying disease progression and primary pathology were not reviewed in the study. It must also be noted that the eventual study number following exclusion is quite low and affects the power of the conclusions. A note also has to be made that this study is in a low-resource, out-of-pocket healthcare financing setting where investigations can be limited by patient factors.

## Conclusions

Tracheostomy care at Wellington Clinics Abuja, Nigeria has significantly improved and the consequence, better patient outcomes, is apparent as shown in this study. We are hopeful this trend is sustained and even further improved as we increasingly adhere to global/local best practice standards.

The reuse of single-use supplies and poor hygiene practices contribute to the increasing rate of healthcare-acquired infections in low-resource settings. Single-use supplies rather than compromising will reduce patient morbidity from chest infections, extended hospital stay, and mortality.

## Appendices

Appendix 1: the existing practice of tracheostomy care

· Inconsistency, poor knowledge, and inadequate training in expected standards for tracheostomy care.

· Inadequate and poor handwashing technique and use of alcohol gel.

· Lack of adequate stoma care.

· Inadequate suctioning of secretions.

· Suction catheter reuse and unhygienic storage.

· Unhygienic patient environment at the bedside.

Appendix 2: practice recommendations

The following recommendations were made, adherence monitored, and a timeline of one year was chosen to assess the impact of our interventions.

· Ensure separate suction materials for suctioning the airway and the mouth.

· Every tracheostomy kit should have an inner tube in place.

· Ensure the airway suction catheter is NOT reused.

· Ensure patients with tracheostomy tubes get the stoma cleaned daily and dressed with povidone dressing.

· Gauze straps should be changed regularly and washed.

· Ensure suction machines are properly connected and cleaned.

· Conducting regular education/training and retraining in tracheostomy care protocol.

· Improved hygiene practices, including hand hygiene, regular audits, and monitoring.

· Early chest radiographs in all ICU patients in whom chest infections or sepsis with chest focus is suspected.

Handover Checklist and Care Protocol

· Tracheostomy supplies for tracheostomy tubes (disposable suction catheters x 5, inner tube present at patient bedside, Yankauer suction catheter, functioning suction machine, manual resuscitation bag, tracheostomy cleaning kit, 10 ml syringes, tracheostomy holder and ties, normal saline, and oxygen source).

· Suctioning frequency for the shift (per hour, two hourly, etc.).

· Secretions: type, amount, colour, and odour.

Appendix 3: indications for suctioning

· Audible and visible secretions build up.

· Reduction in oxygen saturation.

· Diminished breath sounds on auscultation.

· Increased work of breathing.

· To obtain a sputum/nasopharyngeal sample.

· Onset of dyspnea.
